# The Emerging Landscape of Long Non-Coding RNAs in Wilms Tumor

**DOI:** 10.3389/fonc.2021.780925

**Published:** 2022-01-19

**Authors:** Qiang Liu

**Affiliations:** Department of Urology, Cancer Hospital of China Medical University, Liaoning Cancer Hospital and Institute, Shenyang, China

**Keywords:** wilms tumor, long noncoding RNAs (lncRNAs), endogenous competing RNAs (ceRNAs), prognosis, therapeutic targets

## Abstract

Long noncoding RNAs (LncRNAs) are transcripts of nucleic acid sequences with a length of more than 200 bp, which have only partial coding capabilities. Recent studies have shown that lncRNAs located in the nucleus or cytoplasm can be used as gene expression regulatory elements due to their important regulatory effects in a variety of biological processes. Wilms tumor (WT) is a common abdominal tumor in children whose pathogenesis remains unclear. In recent years, many specifically expressed lncRNAs have been found in WT, which affect the occurrence and development of WT. At the same time, lncRNAs may have the capacity to become novel biomarkers for the diagnosis and prognosis of WT. This article reviews related research progress on the relationship between lncRNAs and WT, to provide a new direction for clinical diagnosis and treatment of WT.

## Introduction

Wilms tumor (WT) is a common abdominal tumor that accounts for more than 90% of all malignant renal tumors in children (1–3). The incidence of WT is approximately 1/10,000, and the tumor is known to be highly malignant, to grow fast, and to be prone to metastasis (4–8). In most patients, the first symptom typically involves a palpable abdominal mass. Furthermore, some patients tend to exhibit symptoms such as hematuria, fever, urinary tract infection, varicocele, hypertension or hypotension, and anemia (9–11). Surgical resection combined with postoperative chemotherapy and radiotherapy can significantly improve the 5-year survival rate of WT in children (12–14). However, WT treatment still faces several challenges that need to be overcome to improve the effective rate of treatment, such as multi-drug resistance and the frequency of chemotherapy side effects. In addition, our understanding regarding the pathogenesis and the transformation mechanisms of WT remains insufficient, and there is a lack of corresponding effective targeted therapy (15–18). Therefore, it is urgent to identify specific predictors and therapeutic targets for the prognosis of WT.

In the eukaryotic genome, the proportion of protein-encoding genes is very small because approximately only 1.5% of the DNA in the Homo sapiens genome has the ability to perform this specific function (19). In the process of gene expression, DNA follows the principle of base complementary pairing, and is transcribed under the catalysis of RNA polymerase (20, 21). The transcription products are divided into two types according to their protein-encoding ability. One of these two types is RNA that can encode proteins. In contrast, message RNAs (mRNAs), a type of RNA that cannot encode proteins, are collectively referred to as noncoding RNAs (ncRNAs) (22, 23). At present, noncoding RNAs are mainly classified based on their sequence length, and they are divided into small ncRNAs, which have a sequence length of less than 200 nucleotides, and lncRNAs, which have a sequence length of more than 200 nucleotides (24, 25). LncRNAs are mainly in the form of RNA in epigenetic, transcription, and post-transcriptional regulation of gene expression levels (25–28). Most lncRNAs are also catalyzed and transcribed by RNA polymerase II, but their sequences are not highly conserved, their expression abundance is low, and they display strong specificity in tissues and cells (29–32). The mechanism of action of lncRNAs is also relatively complicated and has been continuously explored in recent years. However, some of the known mechanisms include affecting the transcription of the upstream promoter region of the encoding protein gene and interfering with the expression of downstream genes (33), and inhibiting IIIIA polymerase II or mediating chromatin remodeling and histone modification, thus affecting the expression of downstream genes (34). The transcript forms a complementary double-strand that interferes with mRNA shearing, thus forming different forms of shearing (35) and a complementary double-strand with the transcript of the gene encoding protein, while also generating endogenous siRNA under the action of the Dicer enzyme (36), binding to a specific protein to regulate the activity of the corresponding protein (37), acting as a structural component to form a nucleic acid-protein complex with a protein (38) and as a precursor molecule for small RNAs (miRNAs, piRNAs) (39), and also binding to a specific protein to change the cellular location of the protein (40).

At present, many studies have shown that lncRNAs play an indispensable role in tumor occurrence and progression. Lnc-CCLM is downregulated in cervical cancer tissues and is closely related to the lymphatic metastasis of cervical cancer patients (41). Lnc-GAS6-AS1 is downregulated in lung adenocarcinoma tissue, and it has been related to the patients’ clinicopathological characteristics and survival rates (42). Zhao et al. found that the low expression of lncRNA EMX2OS was associated with the poor prognosis of WT subjects (43). In addition, Zhang et al. showed that lncRNA SOX21-AS1 acts as an oncogenic lncRNA in WT. Knockdown of lncRNA SOX21-AS1 inhibits WT cell proliferation and colony formation, and induces cell cycle arrest by upregulating p57 expression (44). Besides, lncRNA also plays an indispensable role in the occurrence and progression of childhood tumors. Kesherwani et al,. have confirmed the lncRNA expression profile in the pediatric MB subgroups and related molecular pathways and the prognostic significance of lncRNAs and unique lncRNAs associated with each MB subgroup were determined and verified (32591022). They identified important lncRNA DELU2, CASC15, LINC01355 and GAS5 exists in each subgroup, which can further explore the functional analysis of different MB subgroups. In addition, they also found that SOX2, PRKCD, and EZH2 are related to the functional network of each subgroup and may be important drug targets. Smith et al. summarize the abnormally expressed ncRNAs in childhood tumors (45). They found that miRNAs and long non-coding RNAs play a key role in the development of these cancers. In addition, their functional contributions and molecular interactions during tumor formation. Baldini et al,. made an overview. The aim is to briefly summarize the latest findings about the involvement of certain lncRNAs in NB disease by focusing on the mechanism of action of certain lncRNAs involved in NB disease and revealing the possible role in the pathogenesis and progression of NB (45). Liu et al. identified a ceRNA network consisting of 38 DElncRNAs, 18 DEmiRNAs and 99 DEmRNAs was established and 7 prognostic-related RNAs by analyzing the TCGA database (46). It was found through analysis that two RNAs were related to clinical staging and organization. The scientific classification is significantly related, and the 7 RNAs may be considered as new prognostic biomarkers and potential therapeutic targets for WT treatment. In addition, the ceRNA regulatory network can provide new strategies for the further study of lncRNA and miRNA in WT.

This article aims to systematically review the latest research progress of LncRNAs in the field of WT, hoping to provide a theoretical basis for LncRNAs as possible biomarkers and potential therapeutic targets for WT in the future.

## Overview of LncRNAs

### Definition and Classification of LncRNAs

In recent years, as the research on ncRNAs continues to heat up, the function and mechanism of lncRNAs have gradually attracted significant attention from researchers. LncRNAs are types of noncoding RNAs with a length of more than 200 nucleotides (26, 47). Similar to mRNA, most lncRNAs are the products of RNA polymerase II transcription that have a polyA tail and a promoter structure. At the same time, there are significant differences between lncRNA and mRNA because lncRNAs mainly exist in the nucleus, and they also have lower expression levels compared to mRNAs (48–50). Besides, lncRNAs are relatively low conserved, and their expression has temporal and spatial specificity (51–53). Traditionally, it is believed that lncRNAs do not have protein-encoding abilities, but recent studies have identified that a small number of lncRNAs can indeed encode small molecule peptides, thereby regulating biological processes.

There are no clear and unified standards for the classification of LncRNAs. LncRNAs can be classified according to their relative positions to the coding genes and can thus be divided into five categories: sense lncRNAs: the transcription direction is the same as that of the neighboring mRNAs; antisense lncRNAs: the transcription direction is opposite to the transcription direction of the neighboring mRNAs; bidirectional lncRNAs, can be transcribed from the same and opposite directions as adjacent mRNAs at the same time; intergenic lncRNAs, produced by transcription between two genes; and intronic lncRNAs, produced by transcription from the intron region of genes (33, 54–56). Furthermore, lncRNAs can be classified according to their respective role, which can be roughly divided into four categories: signal, decoy, guide, and scaffold molecules (32, 57–64).

### Biological Functions of LncRNAs

#### LncRNAs Act as Diagnostic Markers of Diseases

**Figure 1:**
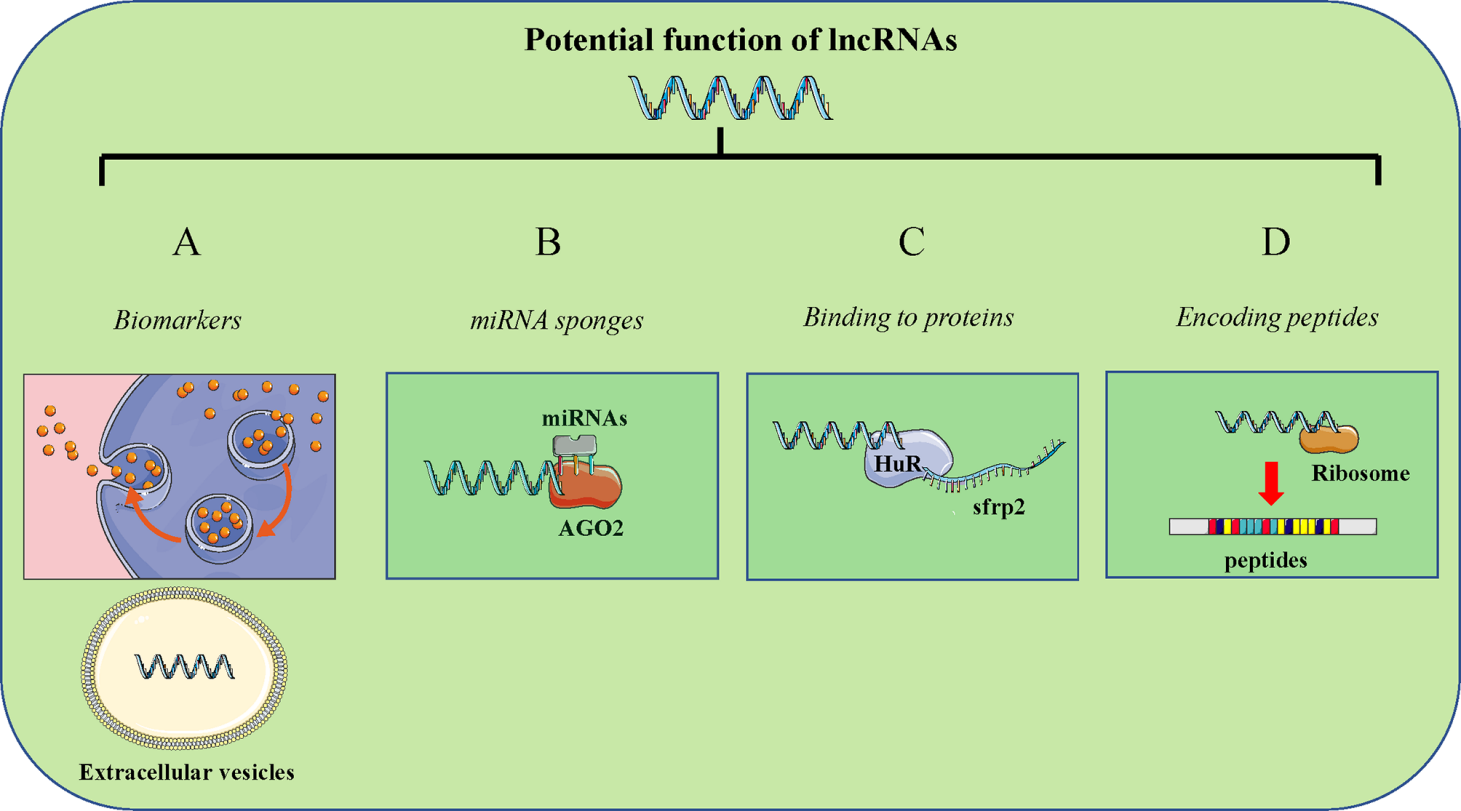
Potential mechanism of lncRNAs in human cancer. **(A)** LncRNAs can act as latent biomarkers; **(B)** LncRNAs can serve as a ceRNA to sponge miRNAs to regulate target genes expression; **(C)** LncRNAs can bind to various proteins (such as transcripition factors); **(D)** LncRNAs have the potential to encode peptides.

The mechanisms of disease occurrence are complex and diverse, mainly due to the abnormal levels of biological macromolecules such as nucleic acids and proteins in the body, which affect normal life activities to varying degrees (65–67). With the advancement of sequencing technology and the emergence of high-throughput sequencing technology, it provides effective help for systematically searching for lncRNAs. At present, through a large number of transcriptome sequencing (RNA-seq), many lncRNAs that are abnormally expressed in diseases have been discovered and identified (68, 69). Compared with normal organisms, lncRNAs with high or low expression must play an important regulatory role in the occurrence of diseases **(** **Figure 1A** **)**. As a diagnostic marker for many diseases. LncRNA DPP10-AS1 is highly expressed in lung cancer tissues, and its up-regulation predicts poor prognosis of patients (70). Lnc-FRLnc1 is significantly up-regulated in the serum and serum exosomes of patients with gastric cancer. The up-regulation of FRLnc1 expression is closely related to lymph node metastasis and TNM staging (71). The high expression of Lnc-SCHLAP1 is significantly related to the adverse clinicopathological characteristics of prostate cancer, including grade group, high pT staging, aggressive cribriform growth/prostatic intraductal carcinoma, and reactive stroma (72).

#### LncRNAs Act as Competitive Endogenous RNA to Regulate mRNA Expression

Competing endogenous RNAs (ceRNAs) are considered to be mRNAs, pseudogenes, and lncRNAs that “communicate” with each other through microRNA response elements (MREs) (73–75). LncRNAs can competitively bind to miRNAs, so that mRNAs can be expressed normally. miRNAs are a type of short-sequence non-coding RNA, which can complement the partial sequence of target mRNAs through MREs, thereby inhibiting the expression of mRNAs (76, 77). There are multiple sites on each mRNA sequence that can bind to miRNAs, which means that one miRNA can bind to multiple mRNAs, or multiple miRNAs can act on the same mRNA. However, as many functions of lncRNAs are gradually being discovered, lncRNAs, as competitive endogenous RNAs, can compete with mRNAs to bind miRNAs through MREs and achieve the effect of protecting the expression of mRNAs **(** **Figure 1B** **)**. Lnc-APF can inhibit autophagy and myocardial infarction by regulating the expression of ATG7 in combination with miR-188-3p (78). The expression of Lnc-AGAP2-AS1 is increased in lung cancer cells and tissues from radiation-sensitive and radiation-resistant patients, and is closely related to the patient. M2 macrophage-derived exosomes AGAP2-AS1 promotes NOTCH2 expression by binding to miR-296 To enhance the radiotherapy immunity of lung cancer (79). The expression of LncRNA OXCT1-AS1 is up-regulated in glioblastoma. The high expression of OXCT1-AS1 indicates a poor prognosis. OXCT1-AS1 may be used as the ceRNA of miR-195 to enhance the expression of CDC25A and promote the progression of glioma cells (80).

#### LncRNAs Can Bind to Proteins

Traditionally, gene regulation in eukaryotes generally refers to the interaction between protein and protein or the interaction between protein and DNA to regulate the expression of coding genes (81–83). However, the regulatory network has a new mode of regulation, that is, RNA and protein. The interaction between RNA and DNA. The main function of coding RNA lies in protein coding, and the regulation of ncRNAs in gene expression has been gradually discovered. LncRNA can regulate the activity of various biological macromolecules, and its main mechanism is that a single lncRNA contains multiple modular domains that bind to DNA, RNA and proteins. Among them, the modular pairing of DNA binding and protein interaction is an important mechanism for lncRNA to perform regulatory functions to recruit chromatin modified proteins that regulate gene expression through chemical modification of histones (63, 84). Of course, the regulation of lncRNAs at the transcription level is also an important type of ncRNAs, and there are many ways in the regulation process, such as influencing gene expression by competing for transcription factors or recruiting protein complexes (**Figure 1C**). The expression of LINC00842 is up-regulated in PDAC and is induced by high concentrations of glucose through the transcription factor YY1. LINC00842 binds to acetylated PGC-1α and prevents the deacetylation of acetylated PGC-1α by the deacetylase SIRT1 to form PGC-1α (85). LINC00511 can recruit EZH2 to the PTEN promoter and promote the methylation of the PTEN promoter, which in turn promotes cell proliferation, migration, stem cell and EMT process (86). Lnc-ELF3-AS1 is highly expressed in gastric cancer tissues and indicates a poor prognosis. ELF3-AS1 may regulate the downstream target gene CC motif chemokine 20 by binding to the RNA binding protein hnRNPK to promote cell proliferation, migration and epithelial stroma Transform and inhibit cell apoptosis (87). Lnc-HoxBlinc can be combined with the promoter region of the characteristic gene of NPM1c, through MLL1 recruitment and promoter H3K4me3 modification to control their activation in HoxBlincTg HSPC, and then act as a cancer-promoting factor to cause the malignant progression of acute myeloid leukemia (AML) (88). FOXC1 can promote the transcription of LINC00301 to promote its expression. LINC00301 can be combined with the enhancer of EZH2 to promote the EAF2 promoter associated with ELL protein. EAF2 directly binds and stabilizes pVHL, so down-regulated EAF2 increases the expression of hypoxia-inducible factor 1 α (HIF1α) by regulating pVHL in non-small cell lung cancer (NSCLC) cells. In addition, LINC00301 can also act as a ceRNA against miR-1276 to accelerate the expression of HIF1α in the cytoplasm of NSCLC (89).

#### LncRNAs Can Encode Polypeptides

Previous studies have shown that lncRNAs do not have the ability to encode. In recent years, many studies have found that lncRNAs can encode very short protein or peptide sequences, and these peptides play an important role in life activities **(** **Figure 1D** **)**. Nelson et al. discovered a putative muscle-specific long-chain non-coding RNA that encodes a 34 amino acid peptide and named it dwarf open reading frame (DWORF) (90). DWORF is located on the SR membrane, where it enhances the activity of SERCA by replacing SERCA inhibitors, phospholipids, creatinine, and mymodulin. Xiang et al. proved that LINC-PINT can encode the peptide PINT87aa, which is significantly increased in the HCC cell senescence model induced by hydrogen peroxide (91). Overexpression of PINT87aa induces growth inhibition, cell senescence and reduction of mitochondrial autophagy *in vitro* and *in vivo*. The peptide RPS4XL encoded by Lnc-Rps4l can inhibit the process of RPS6 by binding to RPS6 and inhibiting the phosphorylation of RPS6 at the phosphorylation site of p-RPS6 (Ser240+Ser244). RPS4XL participates in hypoxia-induced PASMC proliferation (92). LINC00998 can encode a small endogenous peptide called SMIM30. SMIM30 promotes hepatocellular carcinoma tumorigenesis by regulating cell proliferation and migration, and its level is related to the poor survival of HCC patients (93). In addition, SMIM30 is transcribed by c-Myc and then drives the membrane anchoring of the non-receptor tyrosine kinase SRC/YES1.

## Biologic Function of LncRNAsin WT

**Figure 2:**
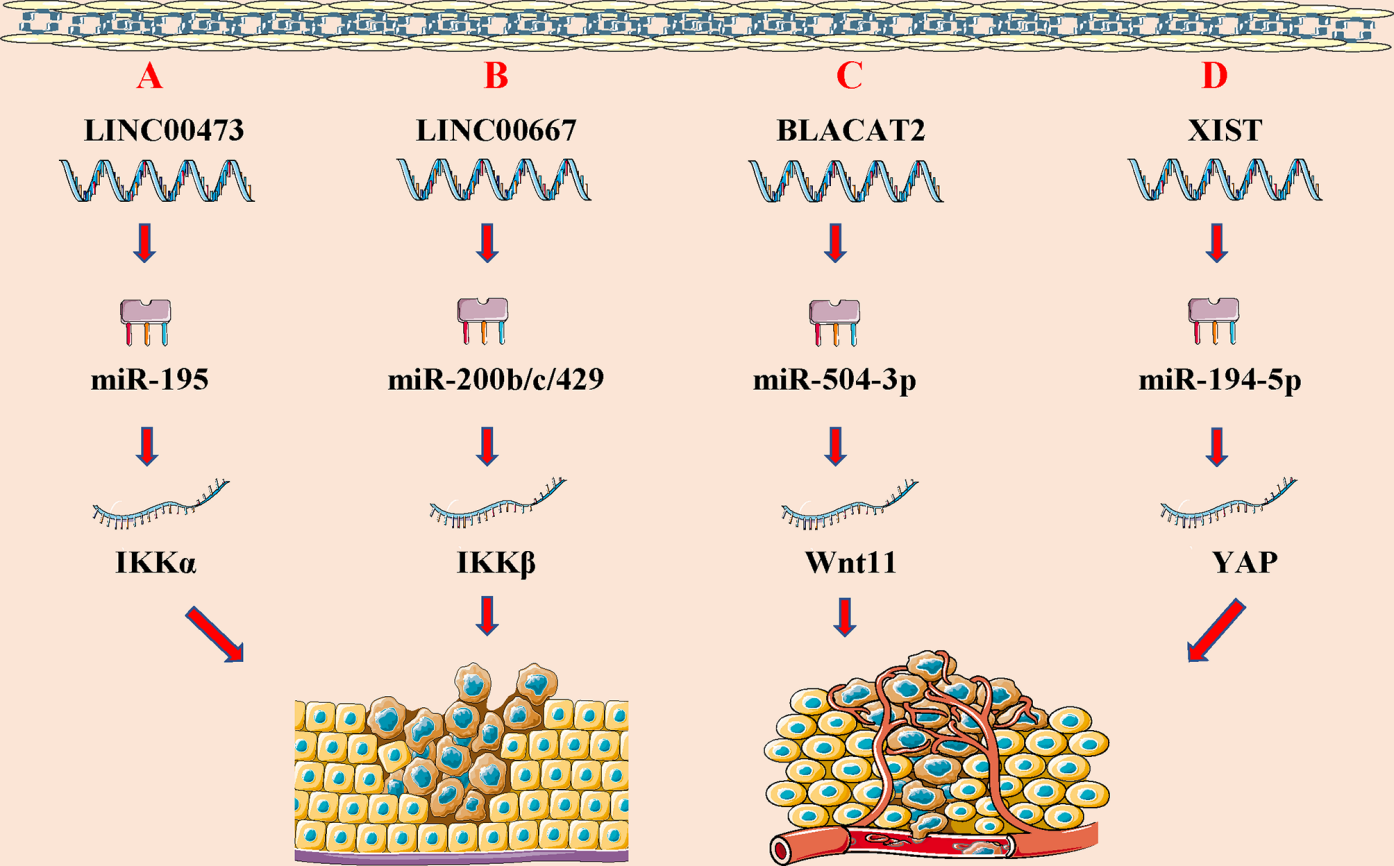
LncRNAs regulate wilims tumor (WT) initiation and progression by acting as ceRNAs. **(A)** lncRNA LINC00473 promotes cell vitality and inhibit cell apoptosis of WT cells by sponging miR-195 and weakening its inhibiting effect on IKKα expression; **(B)** lncRNA LINC00667 regulates the IKKβ expression by sponging miR-200b/c/429, leading to the WT progression; **(C)** lncRNA BLACAT2 functions as the ceRNA to regulate the expression of target gene Wnt11 by sponging miR-504-3p to promote tumor growth and inhibit cell apoptosis; **(D)** lncRNA-XIST influences WT cell growth and metastasis by modulating the XIST/miR-194-5p/YAP axis.

**Figure 3:**
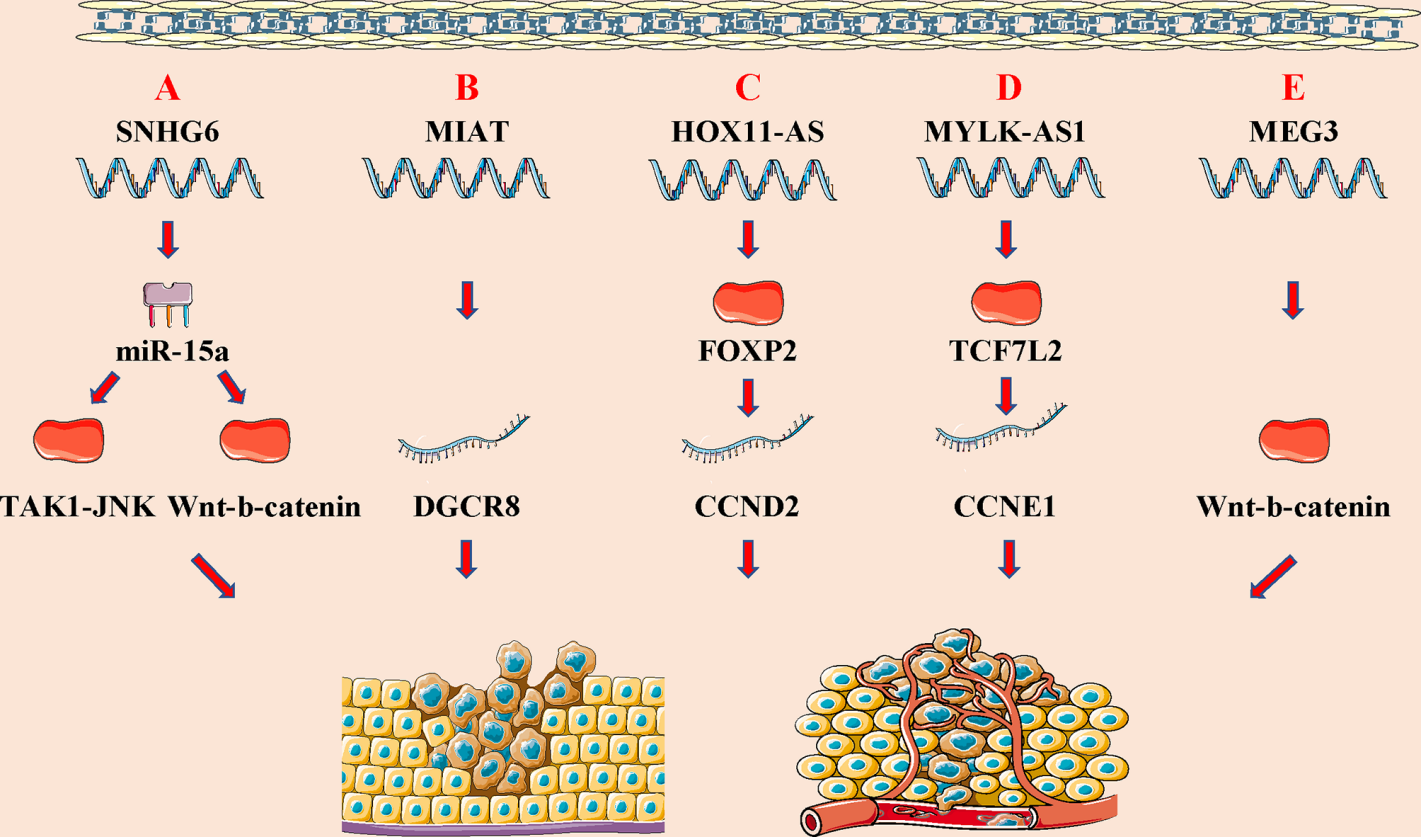
LncRNAs regulate wilims tumor (WT) initiation and progression through modulating distinct signaling pathways. **(A)** lncRNA SNHG6 promote cell proliferation, migration and incursion, and inhibit cell apoptosis of WT by SNHG6/miR-15a/TAK1-JNK and SNHG6/miR-15a/Wnt-b-catenin signaling pathway; **(B)** lncRNA MIAT promote cell growth, migration and invasion of WT by regulating DGCR8 expression; **(C)** lncRNA HOX11-AS inhibits cell apoptosis and promotes cell cycle of WT *via* binding to FOXP2 and regulating CCND2 expression; **(D)** lncRNA MYLK-AS1 promotes cell proliferation and cell cycle *via* binding to TCF7L2 and regulating CCNE1 expression; **(E)** lncRNA MEG3 promotes WT cell growth and metastasis *via* modulating Wnt-b-catenin signaling pathway.

At present, the underlying cause of WT is unclear, and may be related to gene mutations that regulate normal embryonic development in the urogenital tract. Studies have shown that lncRNAs are differentially expressed in WT, which can affect tumor proliferation, invasion, metastasis, and other biological effects through transcriptional and post-transcriptional regulation **(** **Table 1** **)**. LncRNAs can interact with miRNAs through a competitive endogenous RNA mechanism, influence the expression of downstream genes, and thus affect the overall disease process **(** **Figure 2** **)**. LncRNAs can also induce the malignant progression of WT by regulating related pathways **(** **Figure 3** **)**. In addition, lncRNAs have the potential to become tumor markers and potential therapeutic targets in WT **(** **Table 2** **)**.

**Table 1:** Potential role and mechanism of lncRNAs in WT.

LncRNA	Dysregulation	Mechanism	Biological function	Ref.
LINC00473	Up	LINC00473/miR-195/IKKα	promote cell vitality and inhibit cell apoptosis	(94)
				
SNHG6	Up	SNHG6/miR-15a/TAK1-JNK/Wnt-b-catenin	promote cell proliferation, migration and incursion,	(95)
			and inhibit cell apoptosis	
				
MIAT	Up	MIAT/DGCR8	promote cell growth, migration and invasion	(96)
				
HOXA11-AS	Up	HOXA11-AS/FOXP2/CCND2	inhibit cell apoptosis and promote cell cycle	(97)
				
CRNDE	Up	CRNDE/miR-424	promote cell proliferation and metastasis	(98)
				
LINC00667	Up	LINC00667/miR-200b/c/429/IKK-β	promote cell viability, migration and incursion,	(99)
				
XIST	Up	XIST/miR193a-5p	promote cell metastasis	(100)
				
BLACAT2	Up	BLACAT2/miR-504-3p/Wnt11	promote cell proliferation, colony formation,	(101)
			tumor growth and inhibit cell apoptosis	
				
MYLK-AS1	Up	MYLKAS1/TCF7L2/CCNE1	promote cell proliferation and cell cycle	(102)
				
XIST	Up	XIST/miR-194-5p/YAP	promote cell proliferation, migration,invasion	(103)
			and inhibit cell apoptosis	
				
MEG3	Down	MEG3/Wnt/β-catenin	promote cell growth and metastasis	(104)
				

lncRNAs, long nocoding RNAs; WT, wilms tumor; IKKα, component of inhibitor of nuclear factor kappa B kinase complex; SNHG6, small nucleolar RNA host gene 6; TAK1, nuclear receptor subfamily 2 group C member 2; JNK, mitogen-activated protein kinase 8; MIAT, myocardial infarction associated transcript; DGCR8, DGCR8 microprocessor complex subunit; HOXA11-AS, HOXA11 antisense RNA; FOXP2, forkhead box P2; CCND2, cyclin D2; CRNDE, colorectal neoplasia differentially expressed; miRNA, micro RNA; XIST, X inactive specific transcript; Wnt11, Wnt family member 11; MYLK-AS1, MYLK antisense RNA 1; TCF7L2, transcription factor 7 like 2; CCNE1, cyclin E1; MEG3, maternally expressed 3.

**Table 2:** Potential of lncRNAs as dianostic and prognostic tool in WT.

LncRNAs	Source	Detection methods	Biomarker potential	Ref.
MIAT	tissues	qRT-PCR	poor overall survival	(96)
				
XIST	tissues	qRT-PCR	worse prognosis	(100)
				
MEG3	tissues and blood	qRT-PCR	worse prognosis	(104)
				
BLACAT2	tissues	qRT-PCR	worse prognosis	(101)
				
MYLK-AS1	tissues	qRT-PCR	worse prognosis	(102)
				
XIST	tissues	qRT-PCR	worse prognosis	(103)
				

lncRNAs, long nocoding RNAs; WT, wilms tumor; MIAT, myocardial infarction associated transcript; XIST, X inactive specific transcript; MYLK-AS1, MYLK antisense RNA 1; MEG3, maternally expressed 3.

### LncRNAs Regulate WT Growth and Metastasis Through Sponging miRNAs

When lncRNAs act as ceRNAs, it can be combined with miRNAs and “occupy” its MREs. When the content of lncRNAs in the body increases, the bound miRNAs increase, the binding sites of miRNAs and downstream target genes decrease, and the expression of downstream target genes increases. Conversely, when the expression level of lncRNAs in the body decrease, the binding sites on the target miRNAs are empty. In addition, more downstream target genes can be combined with the miRNAs, and the content of downstream target genes that can be detected in the body will be reduced. That is, the expression trends of the two RNAs with the same MRES are consistent. When RNA plays this role, it is also called “molecular sponge”. It is worth noting that there can be multiple RNA binding targets on one miRNA, and one type of lncRNA can also be combined with multiple miRNAs. At this time, more genes are involved and the mechanism is more complicated (105, 106). Studies have found that lncRNA can also be used as ceRNA to regulate the expression of downstream genes in WT. Current research has indicated that LncRNAs could be exploited as competitive endogenous RNAs (ceRNAs) or miRNA sponges. This is because LncRNAs can control the production of target mRNAs at the post-transcriptional level by competitively interacting with miRNAs through their miRNA response elements and reducing miRNA function and activity (106). Zhu et al. found that LINC00473 expression was higher in WT tissues than in normal tissues, and higher levels of LINC00473 have been associated with higher stages and unfavorable histological WT. LINC00473 effectively leads to the occurrence and development of WT through miR-195/IKKα-mediated growth promotion (94). Furthermore, LINC00667 can competitively bind with miR-200b/c/429 to regulate the expression of IKK-β, and subsequently activate the NF-κB pathway in WT, thereby promoting the malignant progression of WT (99). Zhu et al. revealed that the novel BLACAT2/miR-504-3p/Wnt11 axis is related to the occurrence and progression of WT, among which BLACAT2 can absorb miR-504-3p and downregulate Wnt11 (101). The authors also found that the elevated XIST levels in blood and tissue samples of WT patients are significantly related to TNM staging and a shorter survival time. XIST can regulate the miR-194-5p/YAP pathway to promote WT cell proliferation, migration, and invasion, and induce cell apoptosis (103).

### LncRNAs Regulate WT Progression by Regulating Signaling Pathways

**Figure 4:**
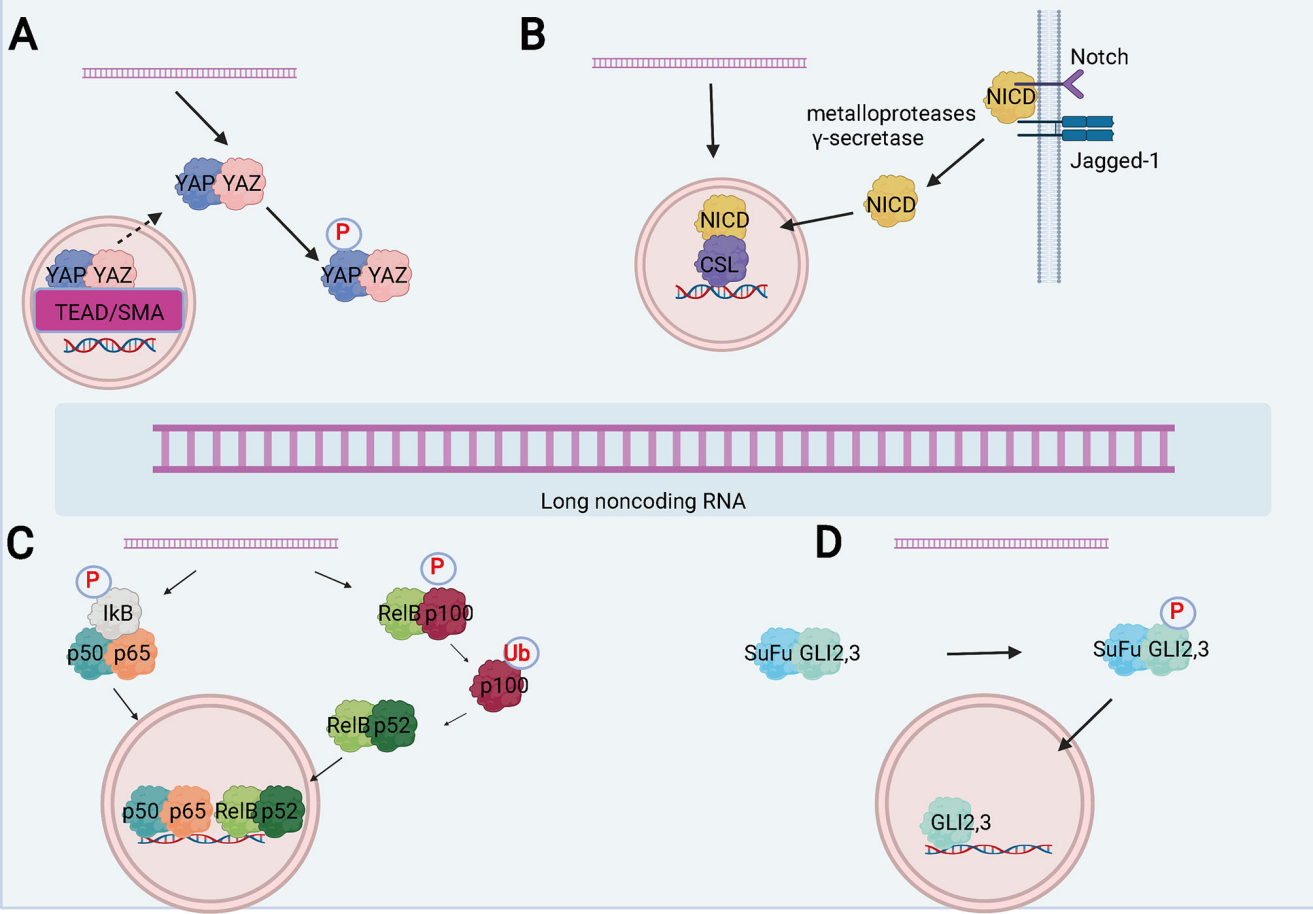
LncRNA can participate in the progression of disease by regulating several signal pathways. **(A)** Schematic of the Hippo signaling pathway and the interaction between lncRNAs and the pathway. **(B)** Schematic of the Notch signaling pathway and the interactions between lncRNAs and the pathway. **(C)** Schematic of the NF-κB signaling pathway and the interactions between lncRNAs and the pathway. **(D)** Schematic of the Hedgehog signaling pathway and the interactions between lncRNAs and the pathway.

Studies have found that lncRNAs can affect the progression of the disease by regulating different signaling pathways (107, 108) **(** **Figure 4** **)**. LncRNA can also participate in cancer progression by regulating different signals in WT. LncRNAs, a class of newly identified genes with gene regulation abilities but no protein-coding capacity, have been proposed to play an important role in regulating physiological processes (109). Su et al. identified that SNHG6 expression is significantly increased in WT tissues. Knockdown of SNHG6 could increase the expression of miR-15a and regulate the TAK1/JNK and Wnt/b-catenin signaling pathways to inhibit cell proliferation, migration, and invasion, and promote cell apoptosis (95). Cui et al. discovered that CRNDE is highly expressed in WT tumor tissues and cell lines, and CRNDE may accelerate the progression of Wilms tumor by regulating microRNA-424 (98). Moreover, Su et al. found that MIAT could promote WT cell proliferation and metastasis by upregulating DGCR8, indicating that MIAT may be a potential target for the diagnosis and treatment of WT (96). Zhu and colleagues found that HOXA11-AS could upregulate the expression of CCND2 *via* recruitment of the FOXP2 transcription factor, thereby inhibiting WT cell apoptosis and promoting cell cycle entry (97). The same group of researchers also found that MYLK-AS1 could promote CCNE1 expression through the TCF7L2 transcription factor, thus regulating cell proliferation and cell cycle distribution, and promoting the tumorigenic ability of WT (102). Liu et al. found that LINC00667 is highly expressed in WT tissues, which can regulate the expression of IKK-β by combining with miR-200b/c/429, thereby inactivating the NF-κB pathway, leading to the malignant progression of WT (99). Furthermore, Teng et al. found that MEG3 is low expressed in WT tissues and blood samples, and can inhibit the proliferation and metastasis of WT cells through the wt/β-catenin pathway (104). Besides, Lyu and colleagues have showed that the low expression of TET2 in WT tissues can further lead to the down-regulation of MEG3 expression, and MEG3 is significantly down-regulated in AML. The down-regulated MEG3 can promote leukemia in a p53-dependent or p53-independent manner (110). Finally, Chen et al. found that lncRNA MEG8 silencing could inhibit the viability, migration, and invasion of WT cells by mediating the miR-23a-3p/CRK axis (111).

### Clinical Significance of LncRNAs in WT

The mechanism of disease occurrence is complex and diverse, mainly due to the abnormal levels of biological macromolecules, such as nucleic acids and proteins in the body, which affect normal life activities to varying degrees (65–67). The advancement of sequencing technology, and the subsequent emergence of high-throughput sequencing technology, has provided effective help for systematically searching for lncRNAs. At present, through a large number of transcriptome sequencing (RNA-seq) datasets, many lncRNAs that are abnormally expressed in diseases have been identified. A variety of lncRNAs have been reported to play an important role in the underlying mechanism of WT. For example, Ren et al. discovered that the characteristics of 3-lncRNA (DLGAP1-AS2, RP11-93B14.6 and RP11554F20.1) signature were significantly correlated with the survival rate of WT patients, and could be used as a prognostic marker for WT patients (112). Liu et al. combined expression data and survival analysis to identify two lncRNAs (HNF1A-AS1 and DELU2) that were significantly related to the overall survival time of WT patients, and may be used as novel prognostic markers for WT (46). Furthermore, Wang et al. found that LINC00087 was significantly related to the overall survival rate of WT patients, and may become a prognostic biomarker of WT (113).

Zhao et al. used qRT-qPCR to detect the expression of MIAT in the tissues of 50 WT patients by dividing patients into two groups based on the median expression of MIAT. The subsequent analysis revealed that patients with high MIAT expression levels had lower overall survival rates than those with low MIAT expression levels (96). Yao et al. analyzed the interaction of XIST expression with WT patients’ age, pathological stage, morbidity, and other indicators, and results showed that the high expression of XIST was positively correlated with the incidence of distant metastasis in WT patients, and was also associated with poor WT prognosis (100). Zhu and colleagues evaluated the potential correlation between the BLACAT2 expression levels and the clinicopathological characteristics of WT patients, and found that there is a strong correlation between high BLACAT2 levels and advanced TNM staging and depth of invasion (101). In addition, high expression of MYLK-AS1 and XIST are both related to the OS rate of WT patients, and high expression of MYLK-AS1 and XIST predicts a poor patient prognosis (102, 103). Finally, Teng et al. found that WT patients with lower MEG3 expression showed more malignant histological types and lymph node metastasis, as well as worse NWTS-5 staging (104).

## Perspections and Conclusion

The discovery of lncRNAs has filled many gaps regarding the molecular mechanism of biological processes. IncRNA performs the functions of signal, scaffold, decoy, and guide molecules. It has also been found that it regulates gene expression from the three levels of transcription, post-transcription, and epigenetics, and it participates in almost all processes of living organisms. However, considering the huge number of lncRNAs and their significant levels of complexity, well-researched lncRNAs are essentially the tip of the iceberg, and there are still many undiscovered functions of lncRNAs. Of course, with the improvement of the current sequencing level and the advancement of research technology, continuously more and more lncRNAs have been found to play important functions in regulating cell cycle, disease occurrence, stem cell differentiation, and cell reprogramming. However, there are many existing challenges. Although more and more lncRNAs have been identified, there are still no unified naming standards. In general, researchers name lncRNAs according to their function and mode of action. However, the definition of lncRNAs is inaccurate. Firstly, not all lncRNAs have a length that is greater than 200 nt, because some sequences annotated as lncRNA can also be less than 200 nt. There are also issues pertaining to the definition of their coding ability because recent studies have found that some lncRNAs have a short open reading frame, which can encode some small peptides.

There are still many problems that need to be overcome, including the means with which we can facilitate early screening for abnormal expression of tumor-related lncRNAs, and intervening, e.g., using related lncRNAs, to improve tumor treatment. Moreover, there are still few studies on lncRNAs in WT. We believe that through continuous in-depth research, lncRNA is expected to become a new opportunity for tumor diagnosis and treatment, and point out a new direction for tumor precision treatment.

In summary, this article reviews the research progress of lncRNAs in WT. Current progress provides new directions and approaches for revealing the molecular mechanism of lncRNAs in WT, and also indicates novel biomarkers for the diagnosis, treatment, and prognosis evaluation of WT.

## Author Contributions

Original draft preparation, allocation, revision, supplement and edition, QL. All authors have read and agreed to the published version of the manuscript.

## Conflict of Interest

The author declares that the research was conducted in the absence of any commercial or financial relationships that could be construed as a potential conflict of interest.

## Publisher’s Note

All claims expressed in this article are solely those of the authors and do not necessarily represent those of their affiliated organizations, or those of the publisher, the editors and the reviewers. Any product that may be evaluated in this article, or claim that may be made by its manufacturer, is not guaranteed or endorsed by the publisher.

## Abbreviations

lncRNAs, long nocoding RNAs; WT, wilms tumor; ceRNAs, endogenous competing RNAs; mRNAs, Message RNAs; snoRNAs, small nucleo-lar RNAs; gRNAs, guide RNAs; snRNAs, small nuclear RNAs; siRNAs, small interfering RNAs; shRNAs, small hairpin RNAs; sdRNAs, sno derived RNAs; piRNAs, Pi-wi interacting RNAs; circRNAs, circular RNAs; IKKα, component of inhibitor of nuclear factor kappa B kinase complex; SNHG6, small nucleolar RNA host gene 6; TAK1, nuclear receptor subfamily 2 group C member 2; JNK, mitogen-activated protein kinase 8; MIAT, myocardial infarction associated transcript; DGCR8, DGCR8 microprocessor complex subunit; HOXA11-AS, HOXA11 antisense RNA; FOXP2, forkhead box P2; CCND2, cyclin D2; CRNDE, colorectal neoplasia differentially expressed; miRNA, micro RNA; XIST, X inactive specific transcript; Wnt11, Wnt family member 11; MYLK-AS1, MYLK antisense RNA 1; TCF7L2, transcription factor 7 like 2; CCNE1, cyclin E1; MEG3, maternally expressed 3.
